# Correction to: Clinical and surgical factors for successful stoma reversal in patients with Crohn’s disease—results of a retrospective cohort study

**DOI:** 10.1007/s00384-024-04594-y

**Published:** 2024-02-02

**Authors:** Clara Ludewig, Veit Jacob, Andreas Stallmach, Tony Bruns, Niels Teich

**Affiliations:** 1 Internistische Gemeinschaftspraxis für Verdauungs- und Stoffwechselkrankheiten, Nordstraße 21, 04105 Leipzig, Germany; 2 Medical Faculty of the Friedrich Schiller University, Jena, Germany; 3 Department of Internal Medicine IV, Jena University Hospital, Jena, Germany; 4 Medical Department III, University Hospital RWTH Aachen, Aachen, Germany

**Correction to: International Journal of Colorectal Disease (2022) 37:2237–2244** 10.1007/s00384-022-04262-z

In this article the affiliation 'Department of Internal Medicine IV, Jena University Hospital, Jena' for Author Clara Ludewig has been added.

The original article has been corrected.

